# Bilateral Lower Cervical Bifurcation of the Common Carotid Artery

**DOI:** 10.1155/2013/894804

**Published:** 2013-07-31

**Authors:** Lokman Uzun, Numan Kokten, Adem Kilicaslan, Bulent Tasel, M. Tayyar Kalcioglu, Muhammet Tekin

**Affiliations:** ^1^Department of ORL, Head & Neck Surgery, Istanbul Medeniyet University, School of Medicine, Goztepe Research and Training Hospital, Dr. Erkin Caddesi, Kadikoy, 34730 Istanbul, Turkey; ^2^Department of Radiology, Istanbul Medeniyet University, School of Medicine, Goztepe Research and Training Hospital, Turkey

## Abstract

Lower cervical bifurcation of cervical common carotid artery (CCA) is a very rarely encountered anatomic variation. Knowing the normal vascular anatomy and also its anomalies is important in preventing the vascular complications. Ill-defined vascular anomalies may lead to massive hemorrhage and eventually death during head and neck surgery. Imaging of the neck by magnetic resonance Imaging (MRI), CT, or angiography is helpful for diagnosis. We present a 62-year-old male patient diagnosed with laryngeal carcinoma who had been treated. His MRI revealed bilateral low-level bifurcation of the cervical common carotid arteries as well as tumor localization and its boundaries. Total laryngectomy and right selective neck dissection was performed to the patient with the diagnosis of squamous cell carcinoma of the larynx. During the neck dissection, carotid bifurcation was detected in common border of Level 3 and Level 4 of the neck.

## 1. Introduction

The neck harbours many vital anatomic structures such as vagus nerve and its branches, carotid arteries, esophagus, and so forth. During the neck surgery, inadvertent injury to these vital structures may cause serious, life-threatening complications. Knowing the normal anatomy and also its anomalies is important in preventing these complications. A wide variety of carotid artery anomalies or anatomic variations which include aplasia, agenesis, tortuosity, hypoplasia, trifurcation, kinked, curved, anomalous origin, independent origins, nonbifurcation cervical carotid artery, and aberrant artery were reported [1–8]. In some rare cases, thoracic bifurcation of the common carotid artery (CCA) may be seen. Newly, Gomez and Arnuk reported the seventh case of the thoracic bifurcation of the common carotid artery [9]. Lower cervical bifurcation of the carotid arteries is a rarely seen anomaly of the carotid artery. Orr first reported the case of the lower cervical carotid artery bifurcation in 1906 [10]. In this paper, we report the case of a 62-year-old man with bilateral low-lying bifurcation of the CCA, and we emphasize the importance of anatomic variations of the carotid artery during the neck surgery.

## 2. Case Report

**Figure 1:**
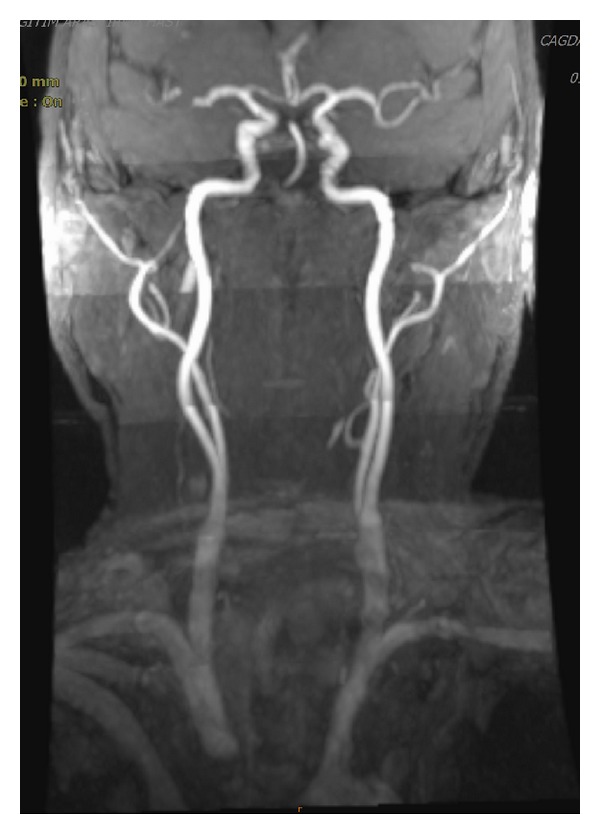
In MR-angiography, 3D ToF image of the patient shows bilateral lower bifurcation of common carotid arteries.

**Figure 2:**
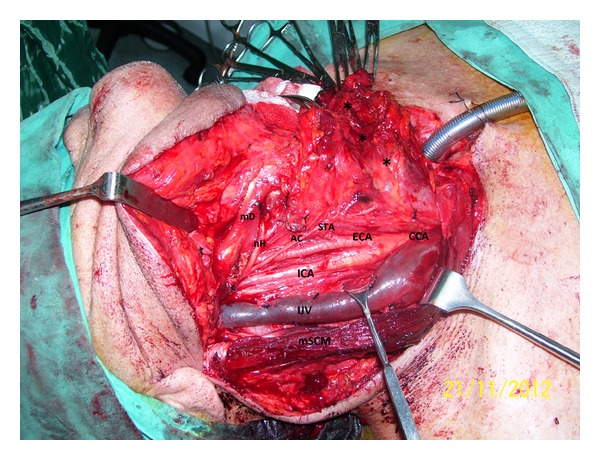
The image shows the lower bifurcation of the right common carotid artery (mD: posterior belly of digastric muscle, nH: hypoglossal nerve, AC: Ansa cervicalis, STA: superior thyroid artery, ECA: external carotid artery, CCA: common carotid artery, ICA: internal carotid artery, IJV: internal jugular vein, mSCM: Sternocleidomastoid muscle, T: tracheostoma, and *: dissection material of the neck spaces—during neck dissection).

We present a 62-year-old male patient who was admitted to our hospital with dysphonia and dyspnea for three months. Laryngeal endoscopy revealed ulcerovegetans tumor on right true and false vocal cord. MRI revealed bilateral low-level bifurcation of the cervical common carotid arteries as well as tumor localization and its boundaries. Three-dimensions time-of-flight (3D ToF) images of MR-angiography showed bilateral lower bifurcation of common carotid arteries (Figure 1). Total laryngectomy and right selective neck dissection was performed to the patient with the diagnosis of T2 N0 Mx squamous cell carcinoma of the larynx. During the neck dissection, carotid bifurcation was detected in common border of Level 3 and Level 4 of the neck (Figure 2). On account of lower cervical bifurcation of CCA, meticulous surgery was performed to avoid any damage to carotid artery and its external and internal branches. Operation and postoperative period were usual. 

## 3. Discussion

The CCAs differ on the right and left sides with respect to their origins. On the right, the common carotid arises from the brachiocephalic artery as it passes behind the sternoclavicular joint. On the left, the common carotid artery comes directly from the arch of the aorta in the superior mediastinum. Following a similar course on both sides, the common carotid artery ascends, diverging laterally from behind the sternoclavicular joint to the level of the upper border of the thyroid cartilage of the larynx (C3-4 junction), where it divides into external and internal carotid arteries. This bifurcation can sometimes be at a higher level [11].

The bifurcation of CCA is most commonly located at the upper level of thyroid cartilage (approximately C4 vertebral level, or common border of Level 2 and Level 3 neck regions), but bifurcation may occur as high as C1 or as low as T4 [12, 13].

Thoracic bifurcation of CCA is a rarely seen anatomic variation. Newly, Gomez and Arnuk reported the seventh case of the thoracic bifurcation of the common carotid artery [9]. Thoracic bifurcation of CCA may be associated with the Klippel-Feil anomaly [12].

Considering the cervical region, Lo et al. [14] found that the CCA bifurcated at the level of the body of hyoid bone in 40%, the superior border of thyroid cartilage in 39%, at the tip of the greater horn of hyoid bone in 15%, and at the body of thyroid cartilage in 6% of the patients in their study which included sixty-seven cadavers.

Ito et al. [15] researched the height of bifurcation of the common carotid artery on cadaver dissection. Bifurcation level between the fourth and fifth vertebra or below the fifth vertebrae accepted as low bifurcation. The high, standard, and low bifurcation rates had been found as 31.2%, 57.5%, and 11.3%, respectively, in that study. In our opinion, high and low bifurcations rates were found relatively high in the studies conducted by Ito et al. [15] and Lo et al. [14], because taken reference points were in a narrow range in the differentiation of high, standard, and low bifurcation. In fact, extreme bifurcation positions are also rarely seen. The frequency of higher cervical bifurcation (C1-C2 level) and the lower cervical bifurcation (C6-C7) was reported as 0.3% and 0.15%, respectively [12]. In our case, bifurcation level was located as low as the boundary of Level 3 and Level 4. Up to date, this case is our first case with extreme bifurcation positions in our neck dissection series reaching several hundred cases.

Lower cervical bifurcation of the carotid arteries was first reported by Orr in 1906. In his case, six mm from its commencement, the right common carotid divides into a large trunk and small trunk. The smaller division was the internal carotid artery [10]. Gulsen et al. [13] reported bilateral low-lying bifurcation of the common carotid artery. In their case, the bifurcation of the CCA was located between the body of the C6 and C7 on the right side and between the body of the C5 and C6 on the left side.

During the neck surgery, the cases with vascular anomalies open to vascular complications. To help minimize operative morbidity and mortality and to reduce the complication rates, a clear understanding of anatomy is essential. Lo et al. [14] found that the origin of the superior thyroid artery appeared to be related to the level of the CCA bifurcation. They reported that presence of high CCA bifurcation should caution surgeons that the hypoglossal nerve is more vulnerable and that the superior thyroid artery may arise from the CCA. Correspondingly, Gulsen et al. [13] had encountered difficulties in a cervical discectomy operation in a patient with low-lying bifurcation of CCA. They had some difficulty because of the lower bifurcation of the CCA, during traction of the tissues to expose the disc spaces and insertion of the cages into the cervical interspaces. In our case, during the neck dissection, carotid bifurcation was detected in common border of Level 3 and Level 4 of the neck (C6-C7 level). On account of lower cervical bifurcation of CCA, meticulous surgery was performed to avoid any damage to carotid artery and its external and internal branches. Operative and postoperative periods were usual, but only dissection of the carotid fascia took more time than usual during the neck dissection.

## 4. Conclusion

Surgery related with carotid artery is fraught with danger, and it requires the knowledge of the anatomical structure of the patients and meticulous dissection. To help minimize operative morbidity and mortality and to reduce the complication rates, a clear understanding of anatomy is essential.
